# Laser ablation synthesis of metal-doped gold clusters from composites of gold nanoparticles with metal organic frameworks

**DOI:** 10.1038/s41598-021-83836-3

**Published:** 2021-02-25

**Authors:** Lukáš Pečinka, Eladia Maria Peña-Méndez, José Elías Conde-González, Josef Havel

**Affiliations:** 1 Department of Chemistry, Faculty of Science, Masaryk University, Kamenice 5, 625 00 Brno, Czech Republic; 2 Departamento de Química, Unidad Departamental de Química Analítica, Facultad de Ciencias, Universidad de La Laguna, Avda. Astrofísico Fco. Sánchez, s/n, 38206 La Laguna, Spain

**Keywords:** Mass spectrometry, Nanoparticles, Nanoscience and technology

## Abstract

Metal-doped gold clusters, mainly cages, are receiving rapidly increasing attention due to their tunable catalytic properties. Their synthesis is mostly based on complex procedures, including several steps. In this work, via adsorption of gold nanoparticles (AuNPs) from aqueous solution to MOF (metal organic frameworks) of M = Co, Cu, Ni, and Zn with various linkers the {AuNPs, MOF} composites were prepared. These composites were used for laser ablation synthesis (LAS) using a common mass spectrometer. Several series of positively and negatively charged Au_*m*_M_*n*_^+/−^ clusters were observed in mass spectra and their stoichiometry (*m* = 1–35, *n* = 1–5) was determined. For each dopant (Co, Cu, Ni, and Zn) ~ 50 different clusters were identified in positive, as well as in negative ion modes. About 100 of these clusters were proposed to be endohedral metal-doped gold cages (for *m* > 12). The developed approach represents a simple procedure for generating metal-doped gold clusters or endohedral metal-doped gold cages materials with potential applications in medicine and/or electronics.

## Introduction

The discovery of doped gold clusters has increased attention to studying the catalytic effect of gold nanoparticles (AuNPs) due to their potential tunable properties^1–5^. The introduction of a metal-dopant atom in the gold clusters changes their electronic, optical, and catalytic properties; thus, metal-doped gold clusters have become an important line of research^1,6–9^. Based on the density functional theory (DFT) and photoelectron spectroscopy (PES), the formation of stable cage-like gold clusters with internal space near to that of fullerenes was evidenced^10^. Internal space in gold cages enables the encapsulation of foreign metals^2,3^. The first stable endohedral-doped gold cages were predicted using DFT and experimentally demonstrated using PES for the W@Au_12_ and Mo@Au_12_ molecules based on the 18-electron rule^11^. Subsequently, an investigation of M@Au_12_ prompted extensive attention to designing novel endohedral-doped gold cages, namely those comprising 12, 14, 15, 16, 17, and 19 gold atoms, for example^1–3,6,12–15^.

The standard approach for clusters generation is based primarily on the laser vaporisation of metal targets (as precursors) in the presence of carrier gas (He); this process is referred to as laser ablation synthesis (LAS)^16–18^. Recently, other precursors have been introduced e.g. metal organic frameworks (MOF)^19,20^. MOF is a broad class of 3D-porous materials with a diverse chemical composition, tunable pore size, and high surface area^21^. MOF can be a host for metal nanoparticles (i.e. AuNPs) to form {AuNPs, MOF} composites^22,23^. AuNP-loaded MOF materials have various applications in catalysis, biomedical applications, and gas storage and separation^21,24^.

MOF composites with adsorbed AuNPs of different size, and/or shape, derivatisation or composition can be used for the LAS of metal-doped gold clusters, as demonstrated for the first time for iron^25^. It was shown that the laser ablation of the {AuNPs, Fe-MOF} composite generates iron-doped gold clusters (containing up to 5 iron atoms). Some of these clusters were suggested to be endohedral iron-doped gold cages. Stabilisation of gold cages via encapsulation of foreign metal atom is well known, and it will be followed in this work^5,7,13,26^. Tunable properties of AuNPs and different MOF compositions show many advantages compared to LAS from metal targets. In this work, we will examine whether this approach can be generalised and used as a standard method for metal-doped gold clusters Au_*m*_M_*n*_^+/−^ synthesis.

## Results and discussion

**Figure 1:**
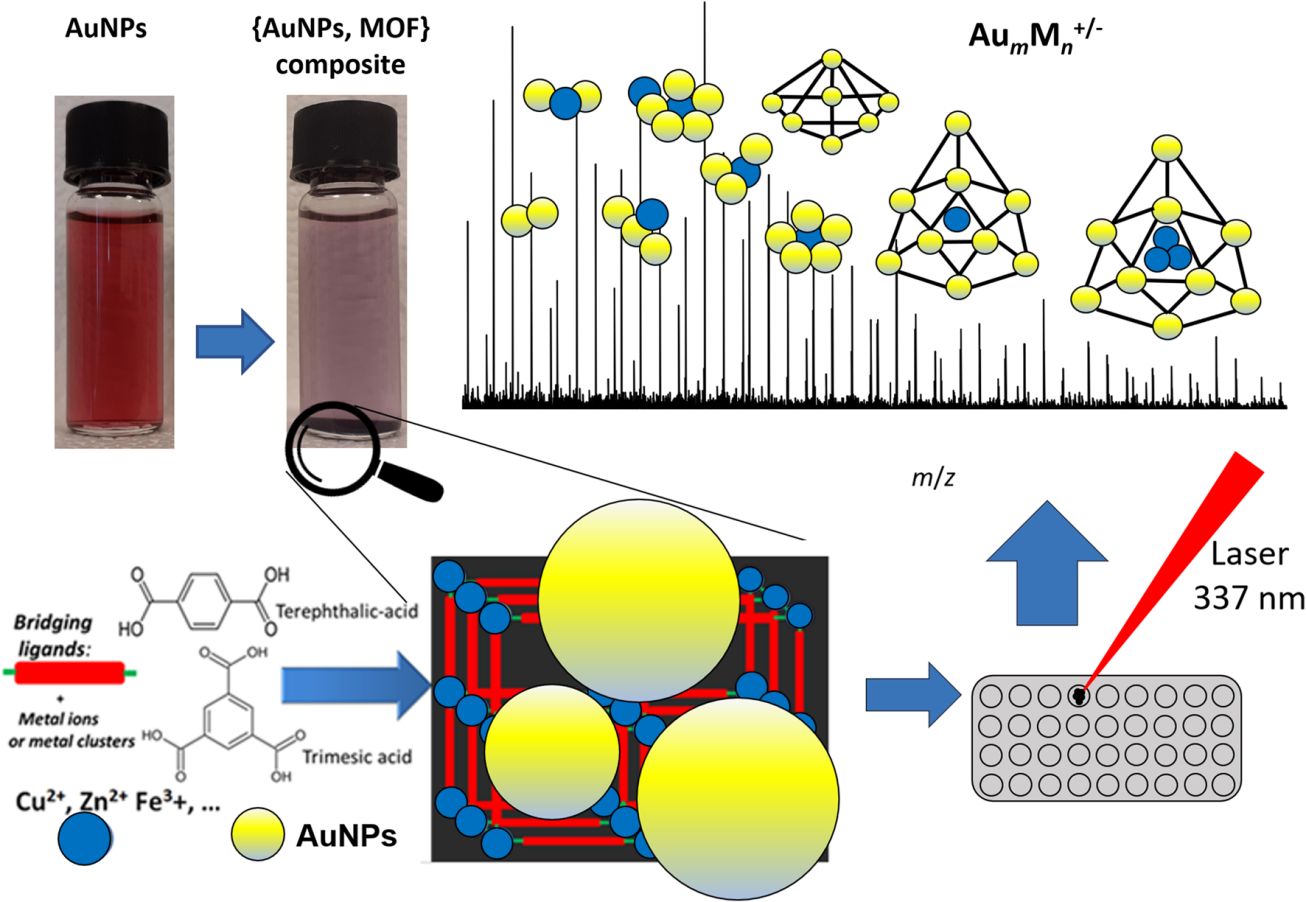
Schematic representation of experimental workflow. Structures have no physical meaning; they are used only for the sake of clarity. The figure was drawn in Microsoft PowerPoint software (version Office365, 2020).

The experimental workflow of the whole procedure is graphically summarised in Fig. 1 and used for all experiments. {AuNPs, MOF} composite (Cu, Co, Ni, and Zn-MOFs) with three different organic linkers (BTC-1,3,5-benzenetricarboxylic, TPA-benzene-1,4-dicarboxylic, and DPA-2,6-pyridinedicarboxylic acids) were prepared using this experimental workflow i.e., examining 12 different systems. A detailed description is given in the Materials and Methods section.

### Laser decomposition of MOF

First, the laser desorption/ionisation (LDI) of selected MOF was studied. An example of a laser-induced decomposition of commercial copper benzene-1,3,5-tricarboxylate is provided in Fig. S1. Similar fragmentation was observed for Co, Ni, and Zn-BTC MOF. Laser decomposition products of MOF were observed in the low mass-to-charge (*m/z*) region and they did not interfere with the clusters of interest (Au_*m*_M_*n*_^+/−^). Moreover, mass spectra indicated fragmentation of the MOF to a low number of organic species, some complexes of metal with fragments of linkers (organic molecules in MOF), and metal clusters.

### LDI of AuNPs and its composites with MOF (Cu, Co, Ni, and Zn)

**Figure 2:**
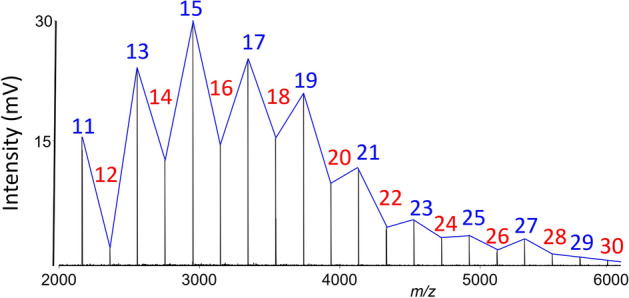
Part of the mass spectrum obtained via laser desorption ionisation of AuNPs where intensities of Au_*m*_^+^ ions correspond to stability, revealing odd–even alternations. Conditions: positive reflectron ion mode, laser energy 130 a.u. Lines connecting maxima/minima in the mass spectrum have no physical meaning; they are used only for the sake of clarity. Blue and red numbers indicate *m* values.

Mass spectra of gold nanoparticles were recorded by applying LDI at a laser energy of 130 a. u. Clusters of Au_*m*_^+^ (*m* = 1–40) were detected (part of the mass spectrum is shown in Fig. 2). Positively single charged gold clusters with an even number of atomic valence electrons (Au_*m*_^+^, *m* = odd) yield higher intensity signals than those with an odd number of electrons. This observation is most likely due to the higher dissociation energy or a lower electron affinity of Au_*m*_^+^ where *m* = odd.

**Figure 3:**
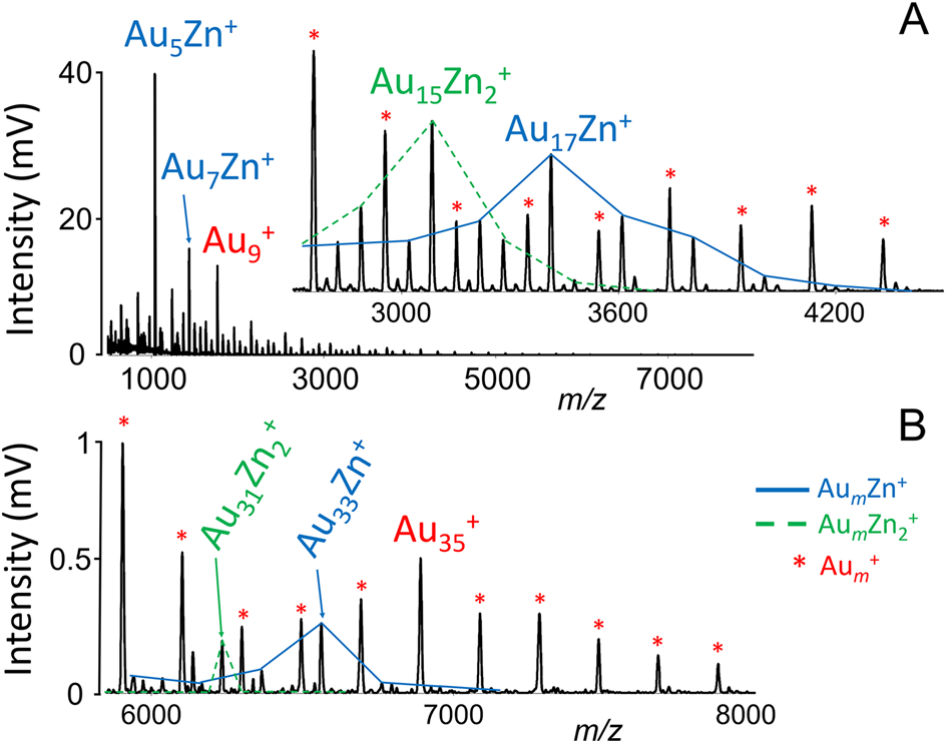
LDI mass spectra of {AuNPs, Zn-MOF} composite showing distribution of Au_*m*_Zn_*n*_^+^ over the *m*/*z* region; gold clusters are marked as ‘*’. Conditions: positive linear ion mode, laser energy 170 a.u. Solid lines have no physical meaning and were used for the sake of clarity.

It is known that this effect is the strongest for small clusters and with increasing numbers of atoms, it gradually disappears and becomes negligible around gold species with *m* = 30 atoms^4,27^. Signals corresponding to gold clusters comprising 8, 18, 20, and 34 itinerant electrons (Au_9_^+^, Au_19_^+^, Au_21_^+^, and Au_35_^+^) are shown in Figs. 2 and 3, where a decrease of signals intensity is observed. The last decrease in intensity of signals in mass spectra was observed for the Au_35_^+^ (34 itinerant electrons) cluster. Figure 3 displays the LDI mass spectra of the {AuNPs, Zn-MOF} composite in positive ion mode. It was observed that the intensity of signals corresponding to the high mass clusters is lower than those detected in the low mass range. For zinc-doped gold clusters, diminishing of signal intensities was observed for the Au_7_Zn^+^, Au_17_Zn^+^ (Au_15_Zn_2_^+^), and Au_33_Zn^+^ (Au_31_Zn_2_^+^) clusters. These cluster sizes match the 8, 18, and 34 itinerant electrons, respectively, if two zinc electrons contribute to the cluster bonding. Doping of gold clusters with copper atoms causes changes in mass spectra intensity at *m*/*z* values corresponding to Au_8_Cu^+^ (Au_7_Cu_2_^+^) and Au_18_Cu^+^ (Au_17_Cu_2_^+^) clusters. These intensity changes correspond to the clusters with key numbers of 8 and 18 itinerant electrons, respectively, assuming that the Cu atom is the donor of one electron. For both elements (Cu, Zn), the electrons originate from 4 s orbitals. The enhanced abundance of the Au_5_Zn^+^ cluster in Fig. 3A indicates a stable structure which agrees with the literature^4^. Ions correspond to charged clusters up to *m*/*z* value 8000 were observed and identified. However, quantum chemistry calculations are required to elucidate the stability of individual Au_*m*_M_*n*_ cluster species, but this is outside the aim of this work.

The effect of laser fluence was studied via recording mass spectra at different laser energies. The threshold laser energy was found at approximately 140 a.u. and the intensity of mass spectra increased to maximal value at laser energy equal to 180 a.u.

The intensities of gold clusters are decreasing at laser energies exceeding 160 a.u, which is due to gold cluster ions decomposition at a higher level of laser energy. In contrast to gold clusters, peaks corresponding to metal-doped gold clusters show higher intensity when the laser energy is increased (exceeding 160 a.u.).

**Figure 4:**
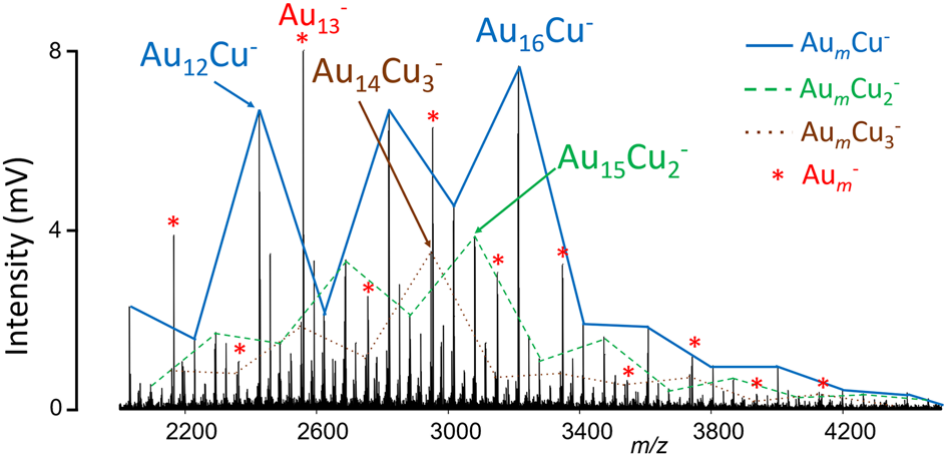
LDI mass spectra of {AuNPs, Cu-MOF} composite showing the formation of Au_*m*_Cu_*n*_^−^ clusters. Conditions: negative reflectron ion mode, laser energy 170 a.u. Solid lines have no physical meaning and were used for the sake of clarity.

All the composite samples were also examined via LDI MS in negative ion mode. Mass spectra measured in this mode gave similar results, as the stoichiometry of most of the negatively charged clusters was the same as in the positive ion mode. Hence, we show only one example. LDI of {AuNPs, Cu-MOF} composite leads to observation of Au_*m*_Cu_*n*_^−^ clusters (Fig. 4). Some oxidised species (Au_*m*_O_1–2_Cu_*n*_^−^) of low intensity were also identified. The Au_16_Cu^−^, Au_15_Cu_2_^−^, and Au_14_Cu_3_^−^ clusters show local maxima of signal intensity in the mass spectra when the so-called 18-electron rule is reached (one e^−^ from each Au or Cu atoms and one due to electron acceptance)^5^. In sum, reaching sufficient laser energy, the reactions in plasma lead to the formation of Au_*m*_M_*n*_^+/−^ clusters (up to *n* = 5).

In addition to 1,3,5-benzenetricarboxylic acid (BTC), MOF with different organic linkers were used. Similar results were obtained for {AuNPs, MOF} composites with TPA (benzene-1,4-dicarboxylic acid) or 2,6-pyridinedicarboxylic acid as organic linker. There are no differences in total number of synthesized clusters or in their stoichiometry.

**Figure 5:**
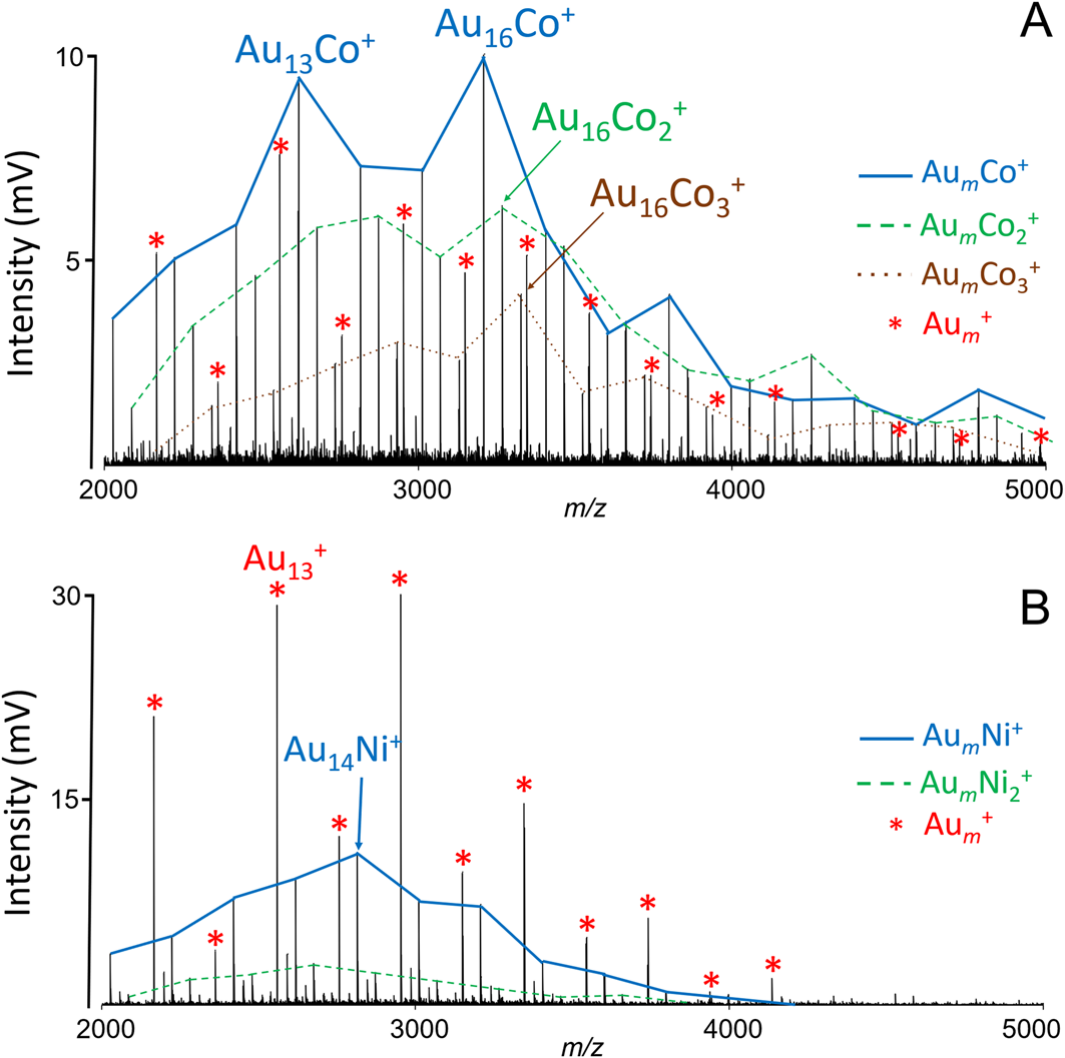
LDI mass spectra of {AuNPs, Co-MOF} (**A**) and {AuNPs, Ni-MOF} (**B**), gold clusters are marked as ‘*’. Conditions: positive reflectron ion mode, laser energy 170 a.u.

Figure 5 shows part of the mass spectra in positive ion mode after performing LDI of {AuNPs, MOF} (M = Co, Ni). The mass spectrum in Fig. 5A shows a series of Au_*m*_Co_1–3_^+^ with Au_16_Co_1–3_^+^ clusters being the most stable ones. On the other hand, laser ablation of {AuNPs, Ni-MOF} composite leads to the formation of only a few Au_*m*_Ni_*n*_^+^ clusters (*n* = 1, 2), most likely due to insufficient laser energy (Fig. 5B).

Doped-gold clusters tend to form 3D structures, and when the number of gold atoms is greater than 9, metal atoms can be encapsulated inside the gold cages. The internal space of gold cages is > 5.5 Å and, therefore, there is space for even more than one atom^10^. Based on our previous article and current work, we are suggesting that up to 5 atoms can be encapsulated inside gold cages^25^. The stoichiometry of detected species was determined via a comparison of experimental and theoretical isotopic distributions (Fig. S2). The most stable and the most intense (in mass spectra) is series of ions where gold clusters are doped with single atom (Au_*m*_M_1_^+/−^). The formation and/or synthesis of Au_*m*_M_*n*_^+/−^ clusters or cages go on most probably in the plasma phase. High excess of AuNPs used in experiments means that AuNPs are surrounding metal atoms coordinated by linker ligands in MOFs structure and such geometry supports the formation of metal-doped gold clusters/cages.

## Conclusions

All selected MOFs adsorb AuNPs from aqueous colloidal solution, forming {AuNPs, MOF} composites while the colour change of red gold nanoparticles changes to dark brown. Laser ablation of the composites leads to the generation of metal-doped gold clusters, detected as positively or negatively single charged ions. In addition to Au_*m*_^+/−^ clusters, mass spectra show the formation of Au_*m*_M_*n*_^+/−^ (*m* = 1–40, *n* = 1–5) clusters. We suggest that gold cages encapsulate 1–5 metal atoms in relation to the gold cages internal space. Conclusively, the proposed method is a useful and simple technique for generating various metal-doped gold clusters. The novelty is that {AuNPs, MOF} composites represent suitable precursors for generating metal-doped gold clusters or endohedral metal-doped gold cages.

## Materials and methods

### Chemicals

Tetrachloroauric acid (HAuCl_4_), gallic acid and copper benzene-1,3,5-tricarboxylate were purchased from Sigma-Aldrich (Steinheim, Germany). All other reagents were of analytical-grade purity. Water was double distilled from a quartz apparatus of Heraeus Quarzschmelze (Hanau, Germany).

### MOF synthesis

Synthesis of MOF materials was based on the slightly modified procedure of Yaghi et al.^28^ Preparation of Zn-BTC MOF: Zn (CH_3_CO_2_)_2_.2H_2_O (0.36 g) was added to 10 ml of an aqueous solution containing 0.2 g of BTC and 0.35 g of NaOH at room temperature, and ultrasound was applied for 30 min. After centrifugation (1000 rpm, 5 min), the precipitate was separated and washed with 10 ml of distilled water. The washing procedure was repeated until the supernatant pH was 7–8. Finally, the precipitate was dried overnight in an oven at 120 °C. For Co-BTC and Ni-BTC, the procedure was the same as described above, also using 0.2 g of BTC while 0.39 g of CoCl_2_ 0.6H_2_O or 0.41 g of Ni(CH_3_CO_2_)_2_ 0.2H_2_O were used.

### Synthesis of {AuNPs, MOF} composites

Adsorption of gold nanoparticles on the MOF was followed using a modified approach as described recently^25^. The AuNPs were generated from HAuCl_4_ (1.6 mL of 1 mM solution) mixed with gallic acid as the reducing agent (2.4 mL of 0.5 mM solution). Gold particles of a colloidal solution with size ~ 50 nm were adsorbed by the MOF under stirring, using a magnetic bar while a distinct colour change from red to dark brown was observed (Fig. 1). The product, {AuNPs, MOF} composite, was collected, washed, dried in an oven below 100 °C, and used for further analysis. Different types of AuNPs were examined. We studied the use of gold nanoflowers (~ 90 nm), several sizes of synthesized colloidal AuNPs, and the use of commercial AuNPs with size < 100 nm^29^. The “best” results (as for the number of metal-doped gold clusters and their intensities) were obtained using colloidal gold with size ~ 50 nm.

### Mass spectrometry

Mass spectra were recorded using either AXIMA Resonance QIT-TOF or AXIMA CFR TOF mass spectrometers (Kratos Analytical, Manchester, UK) equipped with a nitrogen laser (337 nm, frequency 5 Hz, pulse time 3 ns), delayed extraction, and microchannel plate detector. The laser energy was expressed in arbitrary units (0–180 a.u.) while the laser power and fluency at the maximum laser energy were 6 mW and 10 mJ/mm^2^ per one laser pulse. The irradiated spot size was approximately 150 μm in diameter. All measurements were performed in positive or negative linear or reflectron ion mode of detection. The positive and/or negative charge of the clusters was caused by removing and/or accepting one electron. AXIMA Resonance operates in a reflectron ion mode, whereas the AXIMA CFR was used in linear measurements. Mass spectra recorded using AXIMA Resonance were measured in 5 m/*z* ranges: 100–400, 250–1200, 800–3500, 1500–8000, and 3000–15,000. The stainless-steel plate was 2 mm thick with 385 wells, with each sample spot being 2.8 mm in diameter (approximately 6 mm^2^ area). The target plate cleaning was performed according to the Shimadzu target cleaning protocol. Launchpad software (version 2.9.3) from Kratos Analytical (Manchester, UK) was used for interpretation and ion stoichiometry determinations. Gold nanoparticles were used to calibrate mass spectrometers, and the accuracy achieved was below ± 50 mDa.

## Supplementary Information

Supplementary Figures.

## Data Availability

The datasets generated and analysed during the current study are available from the corresponding author on reasonable request.
