# Supplementary material for: Laser ablation synthesis of metal-doped gold clusters from composites of gold nanoparticles with metal organic frameworks
Source: Sci Rep. 2021 Feb 25;11:4656. doi: 10.1038/s41598-021-83836-3 (PMC7907063; doi:10.1038/s41598-021-83836-3)
Supplement: Supplementary file 1 — Supplementary Figures. [file 41598_2021_83836_MOESM1_ESM.docx]

**Supplementary information:**

**Laser ablation synthesis of metal-doped gold clusters from composites of gold nanoparticles with metal organic frameworks**

**Lukáš Pečinka, Eladia María Peña-Méndez, José Elías Conde González, Josef Havel^*^**

***Corresponding author:** havel@chemi.muni.cz (Josef Havel)


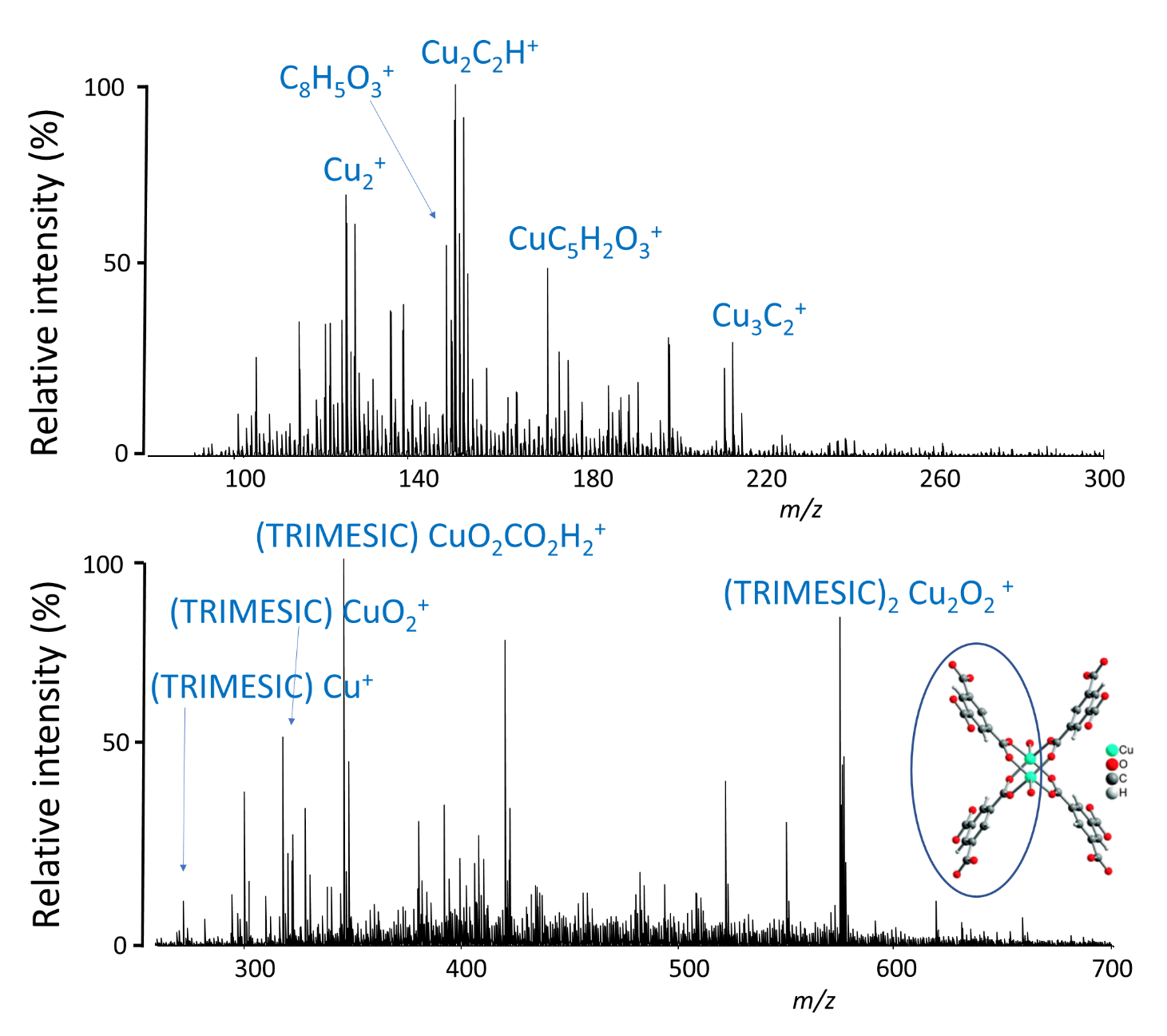


**Figure S1.** LDI mass spectra of Cu-BTC laser-induced decomposition products. Conditions: positive reflectron ion mode, laser energy 120 a.u.


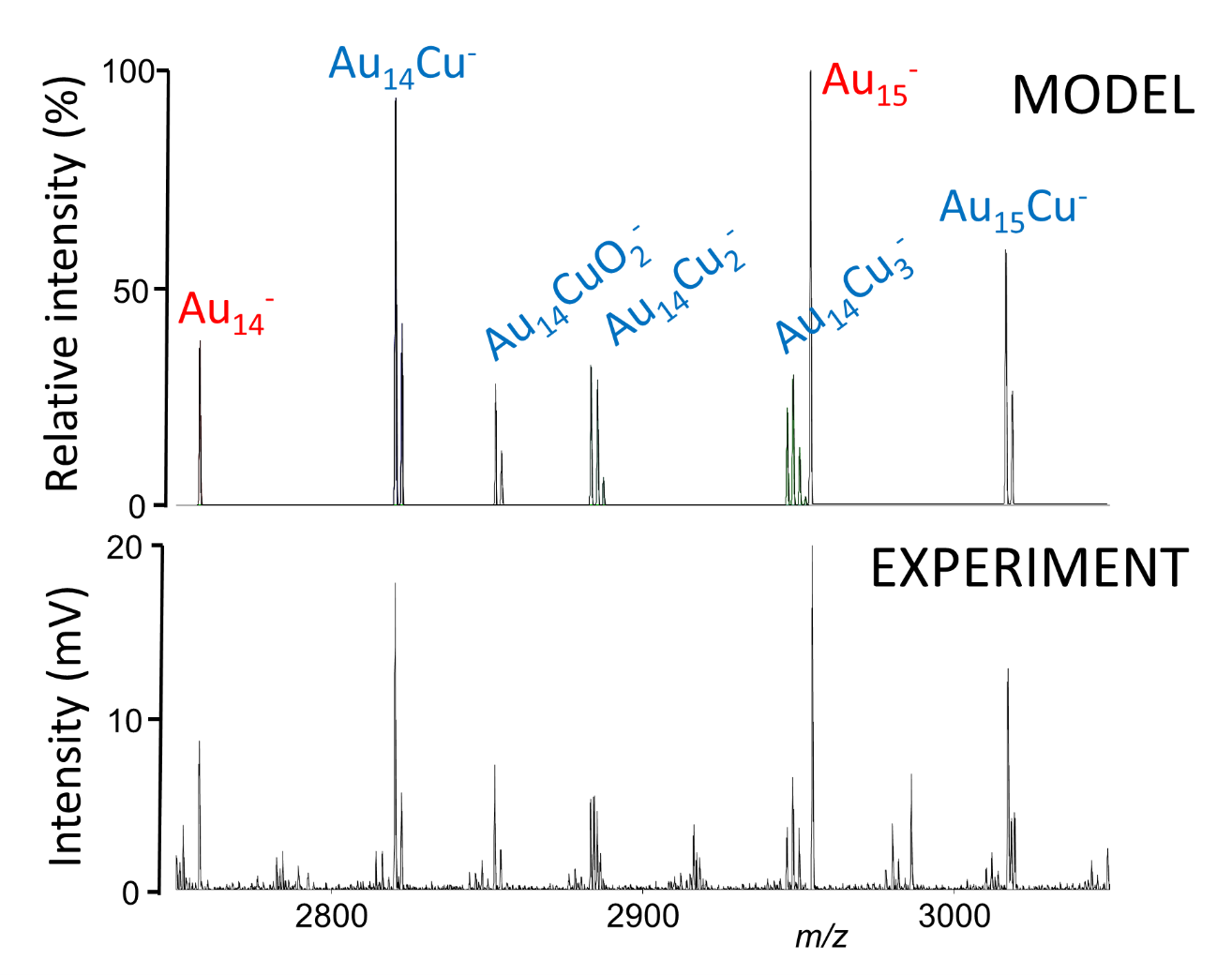


**Figure S2.** Example of experimental mass spectrum of {AuNPs, CuMOF} composite in comparison with theoretical model. Conditions: negative reflectron ion mode, laser energy 170 a.u.
